# Development of a patient decision aid for the management of superficial basal cell carcinoma (BCC) in adults with a limited life expectancy

**DOI:** 10.1186/s12911-020-1081-8

**Published:** 2020-04-29

**Authors:** Alexandra Junn, Neha R Shukla, Lily Morrison, Meghan Halley, Mary-Margaret Chren, Louise C. Walter, Dominick L. Frosch, Dan Matlock, Jeanette S. Torres, Eleni Linos

**Affiliations:** 1 Program for Clinical Research, Department of Dermatology, University of California San Francisco, San Francisco, USA; 2 Department of Dermatology, Stanford University, CCSR Building Room 4235, 269 Campus Drive, Stanford, USA; 3 Palo Alto Medical Foundation Research Institute, Palo Alto, USA; 4 Department of Dermatology, Vanderbilt University Medical Centre, Tennessee, Nashville, USA; 5 Division of Geriatrics, Department of Medicine, University of California, San Francisco, USA; 6 San Francisco and San Francisco VA Medical Center, San Francisco, USA; 7 Department of Medicine, University of California Los Angeles, Los Angeles, USA; 8 Division of Geriatric Medicine, Department of Medicine, University of Colorado School of Medicine, Aurora, USA; 9 VA Eastern Colorado Geriatric Research Education and Clinical Centre, Denver, USA

**Keywords:** Basal cell carcinoma, Shared decision making, Older adults, Decision aid

## Abstract

**Background:**

Basal cell carcinoma (BCC) is a slow-growing, rarely lethal skin cancer that affects people 65 years or older. A range of treatment options exist for BCC, but there is little evidence available to guide patients and providers in selecting the best treatment options.

**Objectives:**

This study outlines the development of a patient decision aid (PDA) for low-risk BCC that can be used by patients and providers to assist in shared decision-making.

**Methods:**

In accordance with the International Patient Decision Aids Standards (IPDAS) Collaboration framework, feedback from focus groups and semi-structured interviews with patients and providers, an initial prototype of the PDA was developed. This was tested using cognitive interviews and iteratively updated.

**Results:**

We created eighteen different iterations using feedback from 24 patients and 34 providers. The key issues identified included: 1) Addressing fear of cancer; 2) Communicating risk and uncertainty; 3) Values clarification; and 4) Time lag to benefit.

**Limitations:**

The PDA does not include all possible treatment options and is currently paper based.

**Conclusions:**

Our PDA has been specifically adapted and designed to support patients with a limited life expectancy in making decisions about their low risk BCC together with their doctors.

## Background

More patients are diagnosed with basal cell carcinoma (BCC) in the US than all other cancers combined, with more than 3.2 million cases of BCC each year, compared to 1.7 million other cancer cases [1]. Over 50% of all skin cancers are diagnosed in patients over 65 and by 2030 this figure is estimated to increase to 70%, notably due to the ageing population [2]. Given the major role of cumulative exposure to ultraviolet radiation from the sun in causing these cancers, most BCCs occur in people 65 years or older [2]. Furthermore, over 100, 000 BCCs are treated in individuals who have a life expectancy of less than a year [3]. In adults with a limited life expectancy (LLE), the risk of treating BCCs may in fact outweigh the benefits. Most BCCs - and specifically those categorized as low-risk - grow very slowly and rarely metastasize (in 0.0029–0.55% of cases) [4]. Given that BCCs cause problems only if left unattended for an extended period of time, it is possible that certain of the more intensive treatment options (i.e., surgical options) may present more of a risk to older patients’ health and quality of life than the BCC itself [3].

Watchful waiting (WW) has been used for other low risk cancers including prostate cancer. In low risk prostate cancer the number of patients choosing WW has increased from 14.5% in 2010 to 42.1% in 2015 [5]. In older adults with a LLE, the option of WW requires consideration of their specific goals and preferences [3]. A method of addressing the unique needs of an older adult with low risk BCC incorporates the use of shared decision making (SDM), which is facilitated by the use of Patient Decision Aids (PDAs) [6]. SDM is defined as the ‘ideal model of treatment decision making in the medical encounter’. Charles, Gafni and Whelan describe SDM as involving 4 components, which we incorporated into our PDA: 1) 2 participants – the physician and patient, 2) both parties share information, 3) both parties are involved in the steps to identify the preferred treatment and 4) a treatment agreement is reached [7].

There are several surgical and non-surgical treatments that are effective in removing BCCs that differ in their side effects. Current clinical guidelines for treatment of BCCs are based primarily on tumor location, size, and histologic type, and do not incorporate patient preferences, or a patient centered treatment approach in older adults and partially tackle life expectancy, frailty and comorbidities [8–11].

PDAs are evidence based tools designed to help patients make specific and deliberated choices among healthcare options [12, 13]. Extensive research has shown that the majority of patients, including dermatologic patients, want to be involved in treatment decisions [14–16]. In situations where multiple treatment options exist, PDAs facilitate patient-centered care by providing both unbiased information about the risks and benefits of various treatment options and tools to help patients consider how their values may align with these treatment options [15]. Studies have shown that older adults are often excluded from making treatment decisions [17]. Furthermore, the underlying health status is pertinent to consider in specifically older adult patients, since this may influence their values and preferences and may determine whether they choose active treatment or WW. A systematic review and meta-analysis of 105 randomized controlled trials found that PDAs increased patient knowledge about management options, reduced decisional conflict, gave patients more accurate expectations about the risks and benefits, and resulted in improved congruence between stated preferences and treatment received [16].

Within dermatology, PDAs exist for psoriasis, melanoma, acne, and oral isotretinoin [18–23]. The existing PDA for BCC developed by Healthwise presents only surgery or medicated creams as options, and does not include WW. The current available decision aid is not specific to older adults with low risk BCC [24]. The PDA described in this paper is aimed at addressing treatment options available to patients with low-risk BCC and limited life expectancy (LLE), for whom WW may be an appropriate treatment option. This paper describes the process of developing a PDA, focusing on decisional aspects unique to BCC.

## Methods

### Overview and conceptual framework

**Fig. 1:**
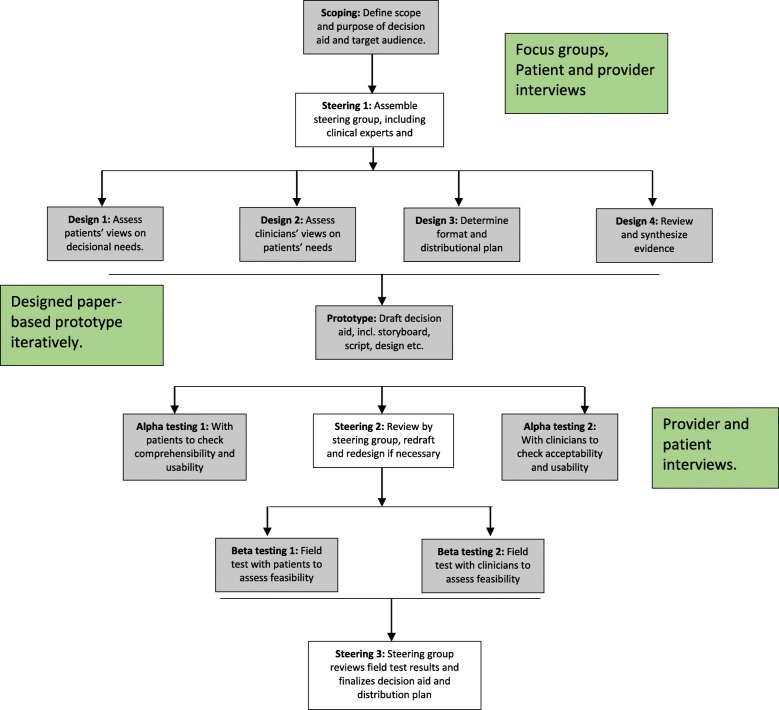
IPDAS Integrated Development Model for Decision Aids

We based our development process on the most recent iteration of the International Patient Decision Aids Standards (IPDAS) Collaboration framework (Fig. 1) [25–28]. The IPDAS process includes five steps; 1) scoping and design; 2) prototype development; 3) “alpha” testing to assess comprehensibility and usability; 4) “beta” testing in the intended clinical environment; and 5) production of a final version. This paper focuses on our methodology for designing, developing, and alpha testing our initial prototype of this PDA.

### Scoping and design

We chose to focus our PDA on patients over the age of 85 with low-risk BCC due to the 1) prevalence of this condition; 2) existence of multiple effective treatment options; 3) unique healthcare needs and challenges for patients with LLE. Our study team included experts in dermatology, geriatrics, cancer in older adults, and shared decision-making.

To design the prototype, we obtained feedback from focus groups and interviews with patients and providers (Table 1). To identify patients, flyers containing the eligibility criteria were posted in public areas of the clinic and distributed by providers. Eligibility criteria included: 1) able to read and speak English; 2) age ≥ 85 years old, OR age 75–79 with a Charlson score of ≥3, OR 80–84 years old with a Charlson score of ≥2 (estimated life expectancy of <5 years); and 3) have a BCC diagnosis in the past 3 years. Interviews were offered to patients who were physically unable to attend a lengthy focus group.

**Table 1:** Numbers of Patient and Providers who gave Feedback during the Development of the Decision Aid

	Patients	Geriatricians	Dermatologists	Total
Phase 1 Design^a^	17	13	15	45
Phase 2 Design^a^	7	0	6	13
Total	24	13	21	58

^a^Phase 1: Developing the prototype of the Decision Aid using focus groups and cognitive interviews

Phase 2: Alpha testing and iterative improvements of the decision aid through patient and provider interviews

Each participant completed a questionnaire collecting socio-demographic characteristics and prior treatments received for skin cancer. A structured focus group/interview guide was used to discuss: 1) individual, social, and structural factors that shape BCC treatment decisions, 2) patient information needs, and 3) overall patient concerns, experiences, and preferences regarding BCC treatment. Finally, the facilitator presented the participant(s) with potential materials for inclusion in the PDA to gather their feedback. These materials were modelled after an existing PDA developed by one of our team advisors [29].

To gather care providers’ perspectives, we identified existing meetings or continuing education opportunities (e.g. grand rounds, works-in-progress meetings, lecture series) typically attended by geriatricians and dermatologists. We then used these forums to present the topic and gather feedback on the PDA materials. As this was part of a public forum, we did not collect individual provider information.

### Development of prototype

Feedback gathered from patients and providers in the design phase was documented using detailed handwritten notes and analyzed using rapid qualitative analysis techniques [30]. We predominantly interviewed the providers with regards to the development of the decision aid, since they were responsible for discussing the diagnosis with the patient and the treatment options. After focus groups or interviews, the research team reviewed these notes and made modifications to the potential PDA materials for review by subsequent participants.

The result of this iterative process was a paper-based prototype of the PDA, which focused on the patients’ decisional needs including the knowledge, expectations, and values related to the decision. The prototype included information on seven different treatments for BCC and the pros and cons of each option. The geriatricians suggested including a modified version of the 10-question Lee Schonberg Index (LSI) which uses health parameters to estimate life expectancy [31, 32]. Because the LSI generates a final score that correlates to life expectancy, we incorporated this score into the treatment comparisons table by suggesting that those with higher scores (lower life expectancy) might be better candidates for less invasive options.

### Alpha testing

To gather feedback on the comprehensibility and usability of the PDA prototype, we conducted cognitive interviews with patients and providers. This technique used a ‘think aloud’ process to ascertain any concerns or lack of clarity in the PDA [33]. Patients were recruited using the same methods and eligibility criteria as above. In addition, we recruited patients who met these eligibility criteria, but did not have a history of BCCs, to approximate the knowledge level of a patient who was newly diagnosed. Patients were interviewed in their homes. The interviewers reviewed the prototype PDA section by section, using think aloud and structured probing techniques to elicit detailed feedback on their reactions to the text and visuals and the overall structure and format of the PDA.

To obtain provider feedback, we identified general dermatologists (as those most likely to use the PDA with their patients) who saw patients at least 2 days per week in one of three settings: academic, community, and private. The study team reached out to individuals directly to conduct the interviews. The cognitive interview guide used during the provider interviews focused on: 1) whether the PDA was a fair representation of low risk BCC, 2) accuracy of information, and 3) logistics of use in clinical practice.

The PDA was revised after each interview, using the same iterative approach used to develop the prototype. This process was deemed complete when interviews with providers offered no significant new suggestions to the existing version.

Throughout the development process, the team also met regularly to discuss and identify emergent themes based on the detailed notes documenting patient and provider feedback. An initial draft of these key issues was developed after the PDA design phase and then collaboratively revised by the study team. The final list of themes described below, were defined as suggestions from participants that were raised at all stages of the development process, specific to the condition of BCCs in adults with a LLE.

## Results

In total we collected feedback from 24 patients and 34 providers, including both general dermatologists (*n* = 21) and geriatricians (*n* = 13) (Table 1). Below we describe four themes relevant to decisional aspects specific to BCC that emerged throughout the development process and how these issues were addressed in each stage of modifications to the PDA. The final version of the PDA is presented in Additional file 1.

### Fear of Cancer

A central issue that arose in the development process was patients’ initial fear upon hearing the word “cancer.” To address this, we initially included the phrase, “The word cancer is scary. Many patients in the same boat have felt afraid or confused,” in an attempt to support the patient’s emotional reaction upon receiving their diagnosis.

However, patients reported that the initial shock at hearing the word “cancer” eventually wore down as they understood how BCCs compared to other cancers. Based on this feedback, we rephrased the sentence: “While the word cancer is scary, yours is a unique kind that does not typically spread or affect how long you will live.” (Additional file 1, Page 4).

### Communicating risk and uncertainty

At the heart of the PDA is the comparison of the treatment options using numbers and visuals that are simple, easy to read and interpret. However, as with many medical treatments, the evidence needed to complete our treatment comparison table is somewhat variable. In addition, there is no data showing the natural history of BCCs, so it was challenging to communicate the numerical risk of WW. Furthermore, statistics that may be important to patients (e.g. length of the procedure) are aspects of clinical practice not necessarily found in the medical literature.

To address these issues, the final table (Additional file 1, Page 5) was iterated by both physicians and patients. We asked a panel of dermatologists at their group meeting to assess the accuracy of the chosen statistics included in the final table. We also asked dermatologists to clarify additional clinical aspects of BCC treatment, including length of the various procedures, recovery times, and timing and intensity of follow-up. We ultimately decided to include the five most common and most well-studied treatments, (Mohs Surgery, Surgery, Scraping Off, Creams, and WW) while excluding two others (Curettage and Electrodessication and Cryotherapy) that were in our prototype. Patients also provided extensive feedback on the format and clarity of the table at each phase.

### Values clarification

Our initial PDA included a survey that was modelled after existing values clarification methods, asking patients to rank the relative importance they placed on value statements that related to a specific treatment option, such as “I want to avoid surgery.” However, patients found this survey confusing, and had difficulty linking survey options to their corresponding treatments.

In response to this feedback, we replaced the survey with process narratives. Using this model, we provided different justifications for the various treatment options (Additional file 1, page 7).

These narratives were designed to allow patients to construct preferences based on hypothetical patients with whom they could identify [34]. Providers also favored the process narratives because they felt that the narratives synthesized the pros and cons of various treatment options in a relatable and understandable way. Thus, for a decision involving multiple treatment options with various trade-offs, sample patient narratives were the preferred method for helping patients clarify their preferences.

### Time lag to benefit

The final issue we addressed in this PDA was that of life expectancy. Due to the slow-growing nature of low-risk BCCs, the immediate risks of certain treatments might outweigh the long-term benefits of those treatments, particularly for patients with LLE. As a result, we wanted to broach the topic of life expectancy in a careful and sensitive manner.

In our first drafts, we directly approached the topic of life expectancy by stating, “Waiting benefits those who may not live long enough to see their skin cancer spread,” as a pro for WW as a treatment option. However, we received feedback from geriatricians that patients respond negatively to hearing about their limited life expectancy.

Initially we incorporated the LSI in the PDA, however, interview participants found the LSI and its application confusing, and often misinterpreted the intended meaning of the score and its relationship to the treatment options. Thus, we ultimately removed the LSI from the final version of the PDA. Clinicians indicated that they would like to access the LSI directly themselves when discussing treatment options with patients to augment the PDA.

### Final versions

Over the entire development process, we created eighteen different iterations. The final paper PDA is written at a maximum reading level of 7th grade according to 3 popular readability tests (SMOG Index, Linsear Write Formula, and Flesch-Kincaid Grade Level), with the treatment comparison table at a 4th grade reading level. The ease of readability was imperative, since it ensured that patients of all educational backgrounds were able to understand and compare the treatment options for low-risk BCC. Because our target population was over the age of 85, we chose to design the PDA as a paper-based handout, accessible online and intended for distribution within the clinic.

## Discussion

This paper outlining the process of developing a decision aid in older adults with a LLE has generalizable lessons for those wanting to build PDAs for other dermatologic conditions in that population. The final version of our PDA was developed according to the IPDAS checklist, outlined in Table 2 and provided information about options for decision making and presented probabilities of outcomes. PDAs have been shown to improve informed decision making and screening behavior, particularly in prostate cancer [35]. WW has been identified as a treatment option for prostate cancer, breast cancer and thyroid cancer. For patients with prostate cancer, where the risks of treatment outweigh the benefits and the tumor is slow growing, patients elect for WW as a treatment option. Within Oncology, particularly for low risk slow growing tumors, more conservative treatments are being considered by the patient community [36]. We found that it was important to address the inherent fear of cancer associated with BCCs, this helped the patients identify trade-offs associated with different treatment options through patient narratives, and indirectly broached the topic of life expectancy. When communicating risk and uncertainty we drew inspiration from existing PDAs that use grid models to allow patients to compare risks and benefits across treatment options [37–39]. In the initial draft, the risks and benefits were communicated using natural frequencies, rather than percentages to present risk [40]. Different data sources present varying rates of recurrence after treatment of BCCs, which made it difficult to select a single number for the table [41]. During the iterative development process and with input from providers, we were able to develop a treatment options grid.

**Table 2:** IPDAS PDA Checklist

IPDAS PDA Components	Criteria	Does our PDA meet the criteria?
**Content**	*Provide information about options in sufficient detail for decision making?*	✓
	*Present probabilities of outcomes in an unbiased and understandable way?*	✓
	*Include methods for clarifying and expressing patients’ values?*	✓
	*Include structured guidance in deliberation and communication?*	✓
**Development Process**	*Present information in a balanced manner?*	✓
	*Have a systematic development process?*	✓
	*Use up to date scientific evidence that is cited in a reference section or technical document?*	✓
	*Disclose conflicts of interest?*	✓
	*Use plain language?*	✓

The existing literature on patient decision-making has illustrated the importance of “values clarification” to help patients integrate their values and preferences into decision-making processes [31]. However, identifying the most effective method to guide patients through this process, particularly for adults with a LLE, required successive rounds of patient and provider feedback. Within our PDA we used process narratives to help patients with ‘values clarification’. Process narratives are sample patient narratives that illustrate the values and preferences involved in health care decisions [42].

The development of this PDA for the treatment of BCCs in older adults provides not only an important tangible product that has a direct clinical application, but also contributes to the field of shared decision-making in general and the development of PDAs in particular. The lessons learnt through this development process may be valuable in creating other PDAs with multiple treatment options for patients with a LLE. Our study describes the development of a PDA, the SUNDAE guidelines added as a supplementary material are used in evaluation studies of PDAs.

Our study has some limitations. First, feedback came from participants from a single location. Although we have used the term WW as a treatment option, we are aware that active surveillance (AS) is also another term that can be used, that has a related but distinct meaning, further research on this topic will help determine whether AS or WW is the appropriate term [36]. Patients were predominantly white and may not represent regional or social differences in culture and medical decision making. Furthermore, our PDA does not include treatment options rarely used in our patient population, such as cryotherapy and radiation, which future versions of this PDA may need to include as trends change. Finally, our PDA is currently paper-based and lacks videos and interactive tools that could be achieved through a future digital interface.

## Conclusion

Our PDA for low risk BCC has been developed collaboratively with the input of patients and providers. Next, we plan to evaluate the instrument in order to assess if it effectively conveys information and supports shared decision making. We are specifically interested in learning whether the PDA improves outcomes related to patient knowledge, engagement, and satisfaction with treatment, and whether it decreases decisional conflict. By adding WW as a potential treatment option, we anticipate that for patients with a LLE, a more patient centered approach can be taken which optimizes their quality of life.

Our long-term goal is to examine whether use of this PDA could reduce perceived side effects of BCC treatment and improve healthcare utilization and skin-cancer related health outcomes. We anticipate that physician buy-in, in the era of ever-increasing pressures on physician schedules, may present a significant barrier. Therefore, we plan to engage the physician community early and frequently to address our common goal of improving the care older adult patients with BCCs receive.

## Supplementary information

**Additional file 1.**

**Additional file 2.** SUNDAE Checklist

## Data Availability

The datasets generated and/or analysed during the current study are not publicly available but are available from the corresponding author on reasonable request.

## Abbreviations

BCC: Basal Cell Carcinoma
PDA: Patient Decision Aid
LLE: Limited life expectancy
LSI: Lee Schonberg Index
IPDAS: International Patient Decision Aids Standards
WW: Watchful Waiting
