# Supplementary material for: Development of a patient decision aid for the management of superficial basal cell carcinoma (BCC) in adults with a limited life expectancy
Source: BMC Med Inform Decis Mak. 2020 Apr 29;20:81. doi: 10.1186/s12911-020-1081-8 (PMC7191775; doi:10.1186/s12911-020-1081-8)
Supplement: Supplementary file 1 — Additional file 1. [file 12911_2020_1081_MOESM1_ESM.pdf]

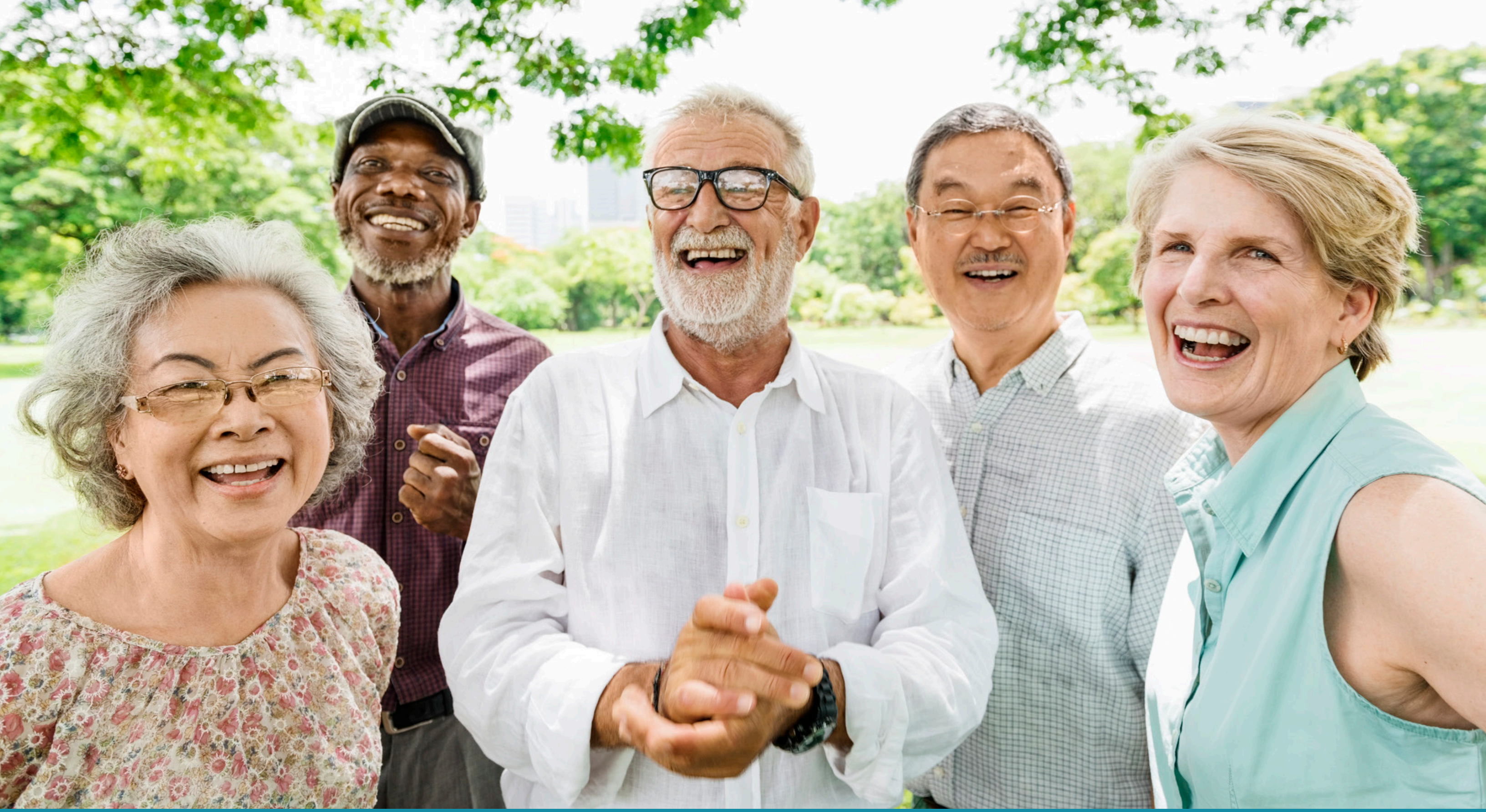

A Decision Aid For The Treatment Of Low Risk Basal Cell Carcinoma

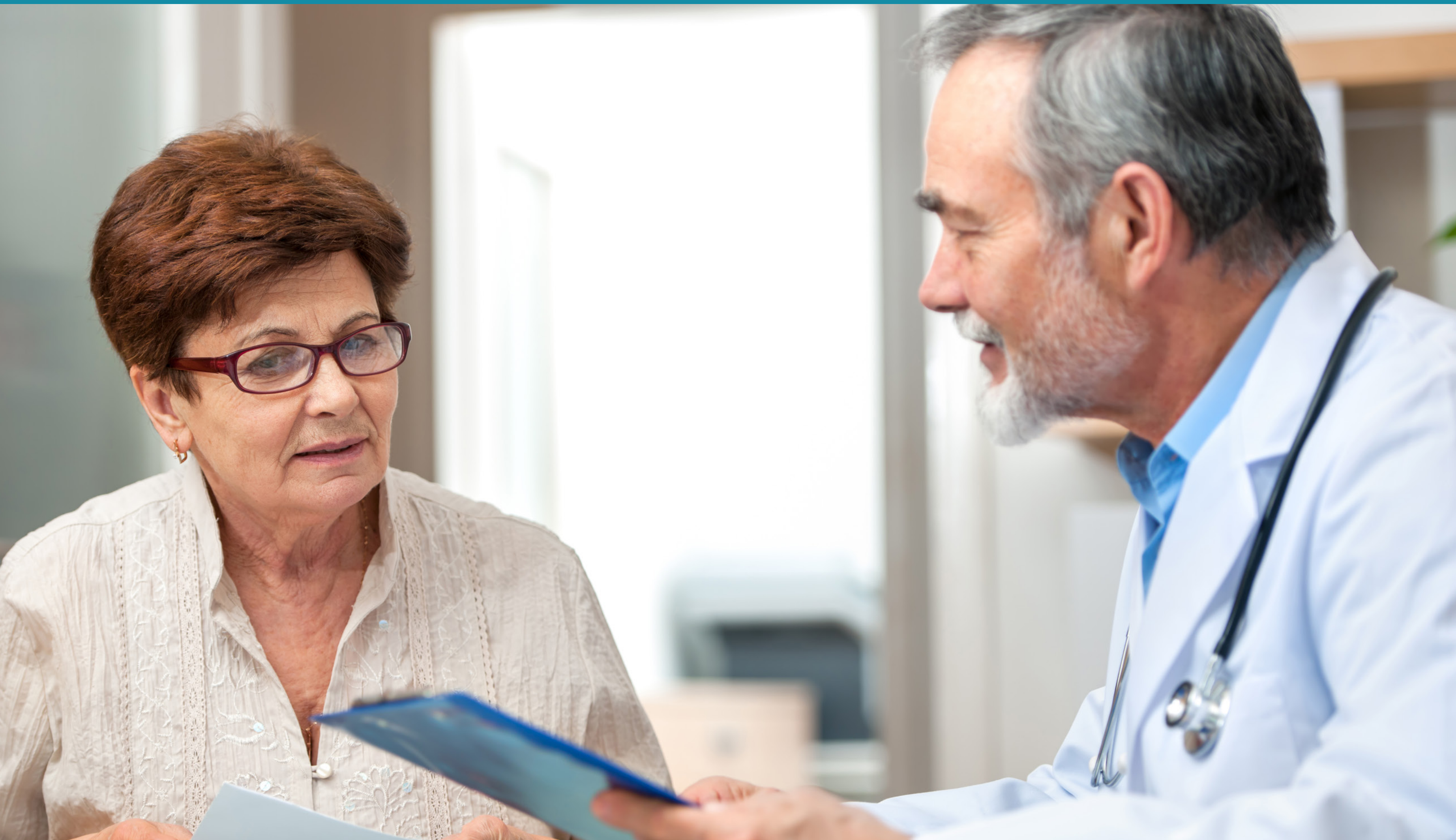

What is a decision aid and why do I need it?

A decision aid is a tool designed to provide information to patients who have multiple options for managing a new diagnosis. There are several treatment choices for patients with low risk BCC, and so your values and preferences are important to determine which treatment option is best for you.

Who is it for?

This decision aid is specifically for patients who have been diagnosed with a low risk basal cell cancer (BCC).

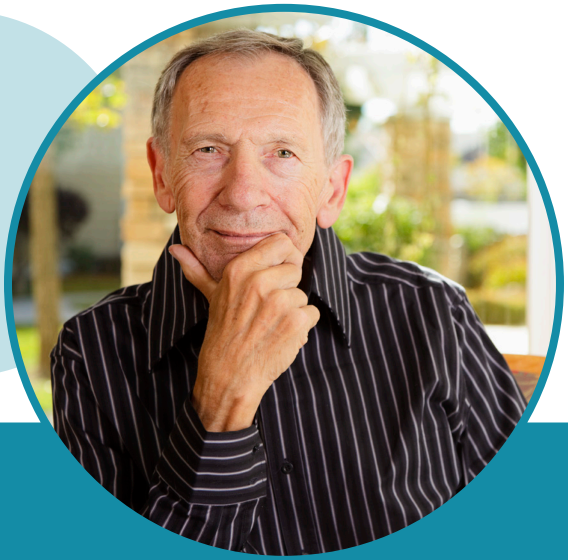

Talk To Your Doctor

Your doctor will be able to tell you which options are available to you, depending on the size and location of your BCC. You should make the right decision for you while talking with your doctor about the risks and benefits of each option. If your doctor does not offer you a choice, and only recommends one treatment, you might ask **"What other ways are there to treat my BCC?"**

While the word cancer is scary, yours is a unique kind that does not typically spread or affect how long you will live.

What is BCC?

Basal cell skin cancer (BCC) is a very common cancer that grows slowly on your skin. It looks like an open sore, red patch or shiny bump. It usually appears on places that have been exposed to the sun.

It is NOT melanoma. Most BCCs are not dangerous. BCCs grow slowly over months and years. BCCs are rarely life-threatening, because only a very small number spread throughout the body.

What is a low risk BCC?

- Less than 2 cm
- Not on the face
- It is not painful, ulcerated or bleeding

What are my treatment options?

There are many different treatments for BCCs including:

- Surgical options
- Non-surgical options
- Watchful waiting

	Mohs Surgery	Surgery
What is it?	Layers of skin are removed and checked under a microscope, until the samples are free of cancer cells. The skin is sewn together with stitches. You will be awake.	The BCC is removed with a thin margin and checked later. The skin is sewn together with stitches. You will be awake.
What is the cure rate?	99%	>98%
What are the risks?	You may experience issues with wound healing, pain, itching, scarring, infection and bleeding.	You may experience issues with wound healing, pain, itching, scarring, infection and bleeding.
How long does it take?	2-8 hours	1 hour
Where does it take place?	At the office	At the office
Will I have a scar?	Yes	Yes
Do I need to come back to remove the stitches?	Yes	Yes
When will I need to come back?	In 1-3 weeks	In 1-3 weeks
How long is the recovery time?	1-3 weeks	1-3 weeks
What happens after the procedure?	You will have to change the bandage everyday for 1-3 weeks, and return to clinic to remove the stitches. Sometimes it takes some time to get back to your daily activities.	You will have to change the bandage every day for 1-3 weeks, and return to clinic to remove the stitches.

Scraping off	Creams	Watchful Waiting
The BCC is scraped and burned three times and a bandage is placed. There are no stitches. You will be awake.	A medicated cream that needs to be applied regularly for 4 to 12 weeks. If you can't reach the site, someone else will need to put it on your skin for you.	The BCC will stay on your skin. If it becomes bothersome to you, you should come back to clinic.
91% to 97%	73% to 83%	Watchful waiting is not a cure.
You may experience issues with wound healing, pain, itching, scarring, infection and bleeding.	You may experience redness, burning or a rash while using the cream.	Your BCC may grow, bleed, become itchy or painful and require larger surgery in the future. Very rarely, it may grow to the point of destroying surrounding tissue.
15 minutes	4-12 weeks	You may reassess at any time
At the office	At home	At home
Yes	Sometimes	No
No	No	No
In 2 weeks-3 months	In 4-12 weeks	In 1-3 months
1-4 weeks	2 weeks after treatment is complete	There is no recovery time
You will have to change the bandage every day for 2-4 weeks.	Your skin will be red and irritated for several weeks.	Your BCC may grow, become itchy or painful and may require a larger surgery in the future.

How do I decide which option is best for me?

Take some time to consider what you have learned about BCC and think about what might be the best treatment for you.

What matters most to you in choosing an option?

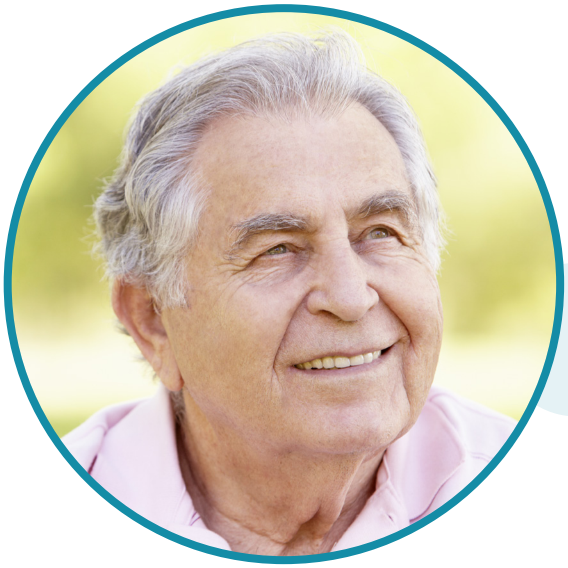

I definitely wanted to have the cancer removed but I liked the idea that this would be a quicker procedure, that did not require an immediate follow up, so I chose scraping off. It's still very effective at removing the BCC, but I didn't need to come back to take out any stitches.

It was important for me to avoid surgery and the risks that came with it, like infection or bleeding, so I chose creams, even though I know the cure rates for creams is a little bit lower and it will take a little bit longer. I really like that there is almost no scar after this treatment.

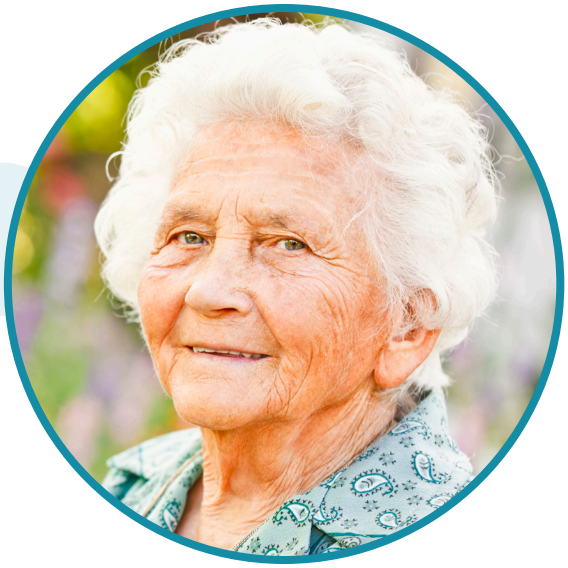

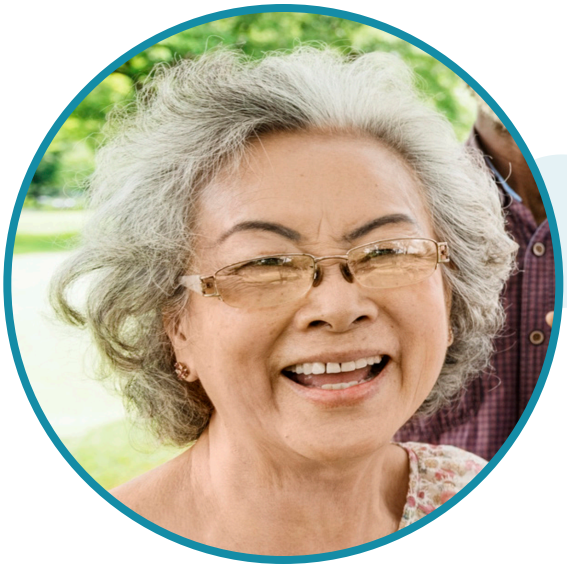

It was really important for me to be as cancer free as possible, and I didn't mind coming back for stitches removal or being in the office for an hour to treat my cancer. So surgery was a good option for me.

I really want to be completely free of my BCC. Therefore, I chose Mohs. It was a longer procedure and I brought someone with me to help me get home.

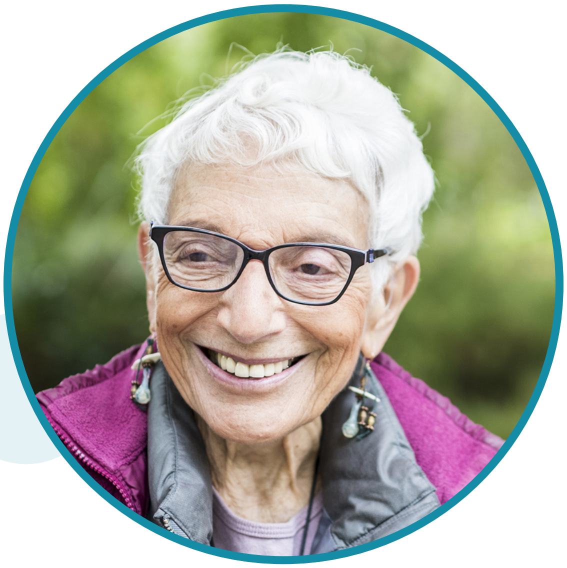

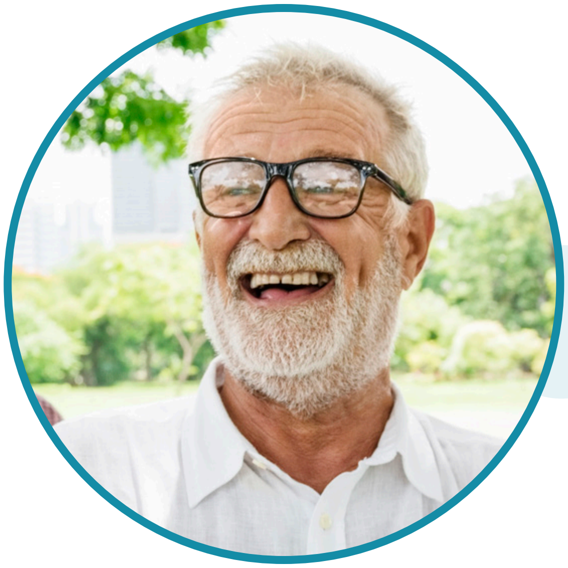

The BCC was so small and didn't bother me, so I didn't even notice it. Therefore, I chose watchful waiting for now. I will keep an eye on it to see if it changes, and I will follow up with my doctor in a few months.

Acknowledgements

We thank the patients, caregivers, and doctors who helped us by giving us advice on this decision tool. Last updated November 6, 2018.

Sources:

1. Bichakjian C, Armstrong A, Baum C, Bordeaux JS, Brown M, Busam KJ, et al. Guidelines of care for the management of basal cell carcinoma. Journal of the American Academy of Dermatology. 2018;78(3):540-59.

2. Chren MM, Linos E, Torres JS, Stuart SE, Parvataneni R, Boscardin WJ. Tumor recurrence 5 years after treatment of cuta- neous basal cell carcinoma and squamous cell carcinoma. J Invest Dermatol. 2013;133(5):1188-1196.

3. Linos E, Schroeder SA, Chren MM. Potential overdiagnosis of basal cell carcinoma in older patients with limited life expectancy. JAMA. 2014;312(10):997-998.

4. Telfer NR, Colver GB, Morton CA. Guidelines for the management of basal cell carcinoma. The British journal of dermatology. 2008;159(1):35-48.

5. Connolly SM, Baker DR, Coldiron BM, Fazio MJ, Storrs PA, Vidimos AT, et al. AAD/ACMS/ASDSA/ASMS 2012 appropriate use criteria for Mohs micrographic surgery: A report of the American Academy of Dermatology, American College of Mohs Surgery, American Society for Dermatologic Surgery Association, and the American Society for Mohs Surgery. Journal of the American Academy of Dermatology. 2012;67(4):531-50.

6. Network NCC. NCCN Guidelines Version 1.2019 Basal cell skin cancer. 2018.

Copyright © 2018
