# Supplementary material for: Development of a patient decision aid for the management of superficial basal cell carcinoma (BCC) in adults with a limited life expectancy
Source: BMC Med Inform Decis Mak. 2020 Apr 29;20:81. doi: 10.1186/s12911-020-1081-8 (PMC7191775; doi:10.1186/s12911-020-1081-8)
Supplement: Supplementary file 2 — Additional file 2. SUNDAE Checklist [file 12911_2020_1081_MOESM2_ESM.docx]

**SUNDAE Checklist**

| **Section Item #** | **SUNDAE Checklist for evaluation studies of patient decision aids** |
| --- | --- |
| **Title/Abstract** |  |
| **1.** | Use the term patient decision aid in the abstract to identify the intervention evaluated and, if possible, in the title. |
| **2.** | In the abstract, identify the main outcomes used to evaluate the patient decision aid. |
| **Introduction** | **As part of standard introduction (the problem, gaps, purpose):** |
| **3.** | Describe the decision that is the focus of the patient decision aid. |
| **4.** | Describe the intended user(s) of the patient decision aid. |
| **5.** | Summarize the need for the patient decision aid under evaluation. |
| **6.** | Describe the purpose of the evaluation study with respect to the patient decision aid. |
| **Methods** | **Studies with a comparator should also address Items 7-13 for the comparator, if possible.** |
| **7.** | Briefly describe the development process for the patient decision aid (and any comparator), or cite other documents that describe the process. At a minimum, include the following:   - Participation of stakeholders in its development - The process for gathering, selecting and appraising evidence to inform its content - Any testing that was done |
| **8.** | Identify the patient decision aid evaluated in the study (and any comparator) by including:   - Name or information that enables it to be identified - Date and/or version number |
| **9.** | Describe the format(s) of the patient decision aid (and any comparator) (e.g. paper, online, video). |
| **10.** | List the options presented in the patient decision aid (and any comparator). |
| **11.** | Indicate the components in the patient decision aid (and any comparator) including:   - Explicit description of the decision* - Description of health problem* - Information on options and their benefits, harms, and consequences* - Values clarification (implicit or explicit)* - Numerical probabilities - Tailoring of information or probabilities - Guidance in deliberation - Guidance in communication - Personal stories - Reading level or other strategies to help understanding - Other components   *These components are needed to meet the definition of a patient decision aid. |
| **12.** | Briefly describe the components from Item 11 that are included in the patient decision aid (and any comparator) or cite other documents that describe the components. |
| **13.** | Describe the delivery of the patient decision aid (and any comparator) including:   - How it was delivered (e.g. by whom and/or by what method) - To whom it was delivered - Where it was used - When it was used in the pathway of care - Any training to support delivery - Setting characteristics and system factors influencing its delivery |
| **14.** | Describe any methods used to assess the degree to which the patient decision aid was delivered and used as intended (also known as fidelity). |
| **15.** | Describe any methods used to understand how and why the patient decision aid works (also known as process evaluation) or cite other documents that describe the methods. |
| **16.** | Identify theories, models or frameworks used to guide the design of the evaluation and selection of study measures. |
| **17.** | For all study measures used to assess the impact of the patient decision aid on patients, health professionals, organization, and health system:   - Identify the measures - Indicate the timing of administration in relation to exposure to the patient decision aid and health care interventions |
| **18.** | For any instruments used:   - Name the instrument and the version (if applicable) - Briefly describe the psychometric properties, or cite other documents |
| **Results** | **In addition to standard reporting of results:** |
| **19.** | Describe the characteristics of the patient, family, and carer population(s) (e.g. health literacy, numeracy, prior experience with treatment options) that may affect patient decision aid outcomes. |
| **20.** | Describe any characteristics of the participating health professionals (e.g. relevant training, usual care vs. study professional, role in decision making) that may affect decision aid outcomes. |
| **21.** | Report any results on the use of the patient decision aid:   - How much and which components were used - Degree to which it was delivered and used as intended (also known as fidelity) |
| **22.** | Report relevant results of any analyses conducted to understand how and why the patient decision aid works (also known as process evaluation). |
| **23.** | Report any unanticipated positive or negative consequences of the patient decision aid. |
| **Discussion** | **As part of the standard discussion section (summary of key findings, interpretation, limitations and conclusion):** |
| **24.** | Discuss whether the patient decision aid worked as intended and interpret the results taking into account the specific context of the study including any process evaluation. |
| **25.** | Discuss any implications of the results for patient decision aid development, research, implementation, and theory, frameworks or models. |
| **Conflict of Interest** | |
| **26.** | All study authors should disclose if they have an interest (professional, financial or intellectual) in any of the options in the patient decision aid or a financial interest in the decision aid itself. |
